# Supplementary material for: Sex-Specific Associations of Red Meat and Processed Meat Consumption with Serum Metabolites in the UK Biobank
Source: Nutrients. 2022 Dec 14;14(24):5306. doi: 10.3390/nu14245306 (PMC9782977; doi:10.3390/nu14245306)
Supplement: Supplementary file 1 [file nutrients-14-05306-s001.zip › nutrients-2068327-supplementary.pdf]

Supplemental Table S1. Baseline characteristics of participants included in the study

	Men<br>(n = 37,963)	Women<br>(n = 41,681)
**Mean processed meat consumption (SD), times/week**	1.89 (1.52)	1.15 (1.17)
**Mean unprocessed red meat consumption (SD), times/week**	2.29 (1.52)	1.98 (1.38)
**Mean age (SD), year**	56.7 (8.2)	56.3 (8.0)
**Ethnic background, %**		
White	94.6	94.6
Asian or Asian British	2.3	1.7
Black or Black British	1.5	1.7
Chinese	0.3	0.3
Mixed	0.4	0.7
Other/unknown	0.9	1.0
**Townsend deprivation index (SD)***	-1.33 (3.04)	-1.25 (3.16)
**With College or University degree education, %**	41	38
**Smoking, %**		
Never	60	49
Previous	31	38
Current	9	13
**Physical activity, %**		
Low	19	19
Moderate	39	43
High	42	38
**Mean BMI (SD), kg/m^2**	27.8 (4.25)	27.1 (5.20)
**Alcohol drinking, %**		
Never	3	6
Previous	4	4
Current	93	90

*Townsend Deprivation Index, higher scores represent higher levels of deprivation

Supplemental Table S2. Sex-specific differences in the associations of unprocessed red meat consumption with metabolites

subgroup	metabolite	zpval
Amino acids	glycine	0.00E+00
Apolipoproteins	apolipoprotein a1	3.95E-02
Branched-chain amino acids	valine	9.30E-03
Branched-chain amino acids	total concentration of branched-chain amino acids (leucine + isoleucine + valine)	3.40E-03
Branched-chain amino acids	leucine	3.00E-04
Branched-chain amino acids	isoleucine	2.89E-02
Chylomicrons and extremely large VLDL	triglycerides in chylomicrons and extremely large vldl	2.43E-02
Chylomicrons and extremely large VLDL	total lipids in chylomicrons and extremely large vldl	4.29E-02
Fatty acids	monounsaturated fatty acids	6.20E-03
Fluid balance	creatinine	4.23E-02
IDL	triglycerides in idl	4.36E-02
Large HDL	total lipids in large hdl	7.00E-04
Large HDL	free cholesterol in large hdl	4.00E-04
Large HDL	concentration of large hdl particles	1.00E-03
Large HDL	cholesteryl esters in large hdl	8.00E-04
Large HDL	cholesterol in large hdl	7.00E-04
Large LDL	triglycerides in large ldl	1.04E-02
Lipoprotein particle sizes	average diameter for hdl particles	9.00E-04
Medium LDL	triglycerides in medium ldl	3.40E-03
Medium LDL	concentration of medium ldl particles	3.80E-03
Medium LDL	cholesteryl esters in medium ldl	3.73E-02
Medium LDL	cholesterol in medium ldl	4.36E-02
Medium VLDL	free cholesterol in medium vldl	4.56E-02
Small LDL	triglycerides in small ldl	8.80E-03
Triglycerides	triglycerides in ldl	7.00E-03
Very large HDL	triglycerides in very large hdl	9.10E-03
Very large HDL	total lipids in very large hdl	1.00E-04
Very large HDL	phospholipids in very large hdl	1.00E-04
Very large HDL	free cholesterol in very large hdl	1.10E-03
Very large HDL	concentration of very large hdl particles	1.00E-04
Very large HDL	cholesteryl esters in very large hdl	2.00E-04
Very large HDL	cholesterol in very large hdl	3.00E-04
Very small VLDL	cholesteryl esters in very small vldl	1.23E-02
Very small VLDL	cholesterol in very small vldl	2.21E-02

Supplemental Table S3. Sex-specific differences in the associations of processed meat consumption with metabolites

subgroup	metabolite	zpval
Amino acids	glycine	0.00E+00
Amino acids	glutamine	2.04E-02
Aromatic amino acids	tyrosine	1.78E-02
Branched-chain amino acids	valine	0.00E+00
Branched-chain amino acids	total concentration of branched-chain amino acids (leucine + isoleucine + valine)	0.00E+00
Branched-chain amino acids	leucine	0.00E+00
Branched-chain amino acids	isoleucine	1.00E-03
Glycolysis related metabolites	lactate	1.43E-02
Inflammation	glycoprotein acetyls	3.00E-04
Large HDL	total lipids in large hdl	0.00E+00
Large HDL	phospholipids in large hdl	0.00E+00
Large HDL	free cholesterol in large hdl	0.00E+00
Large HDL	concentration of large hdl particles	0.00E+00
Large HDL	cholesteryl esters in large hdl	0.00E+00
Large HDL	cholesterol in large hdl	0.00E+00
Large VLDL	total lipids in large vldl	3.06E-02
Large VLDL	phospholipids in large vldl	2.37E-02
Large VLDL	free cholesterol in large vldl	1.81E-02
Large VLDL	concentration of large vldl particles	4.19E-02
Large VLDL	cholesteryl esters in large vldl	2.90E-03
Large VLDL	cholesterol in large vldl	7.00E-03
Lipoprotein particle sizes	average diameter for vldl particles	2.80E-02
Lipoprotein particle sizes	average diameter for ldl particles	1.13E-02
Lipoprotein particle sizes	average diameter for hdl particles	0.00E+00
Medium HDL	free cholesterol in medium hdl	0.00E+00
Medium HDL	cholesteryl esters in medium hdl	0.00E+00
Medium HDL	cholesterol in medium hdl	0.00E+00
Other lipids	phosphoglycerides	3.10E-03
Phospholipids	total phospholipids in lipoprotein particles	3.60E-03
Phospholipids	phospholipids in hdl	0.00E+00
Small HDL	triglycerides in small hdl	5.00E-03
Small VLDL	triglycerides in small vldl	3.56E-02
Small VLDL	total lipids in small vldl	2.03E-02
Small VLDL	concentration of small vldl particles	1.55E-02
Small VLDL	cholesteryl esters in small vldl	8.80E-03

Small VLDL	cholesterol in small vldl	2.14E-02
Total lipids	total lipids in hdl	0.00E+00
Very large HDL	total lipids in very large hdl	0.00E+00
Very large HDL	phospholipids in very large hdl	0.00E+00
Very large HDL	free cholesterol in very large hdl	0.00E+00
Very large HDL	concentration of very large hdl particles	0.00E+00
Very large HDL	cholesteryl esters in very large hdl	0.00E+00
Very large HDL	cholesterol in very large hdl	0.00E+00
Very large VLDL	cholesteryl esters in very large vldl	1.00E-02
Very large VLDL	cholesterol in very large vldl	2.47E-02
